# Air pollution exposure during urban running in Frankfurt, Germany: a randomized crossover comparison of particulate matter levels on park, roadside, and residential routes across morning and early afternoon hours

**DOI:** 10.1186/s13690-026-02137-1

**Published:** 2026-11-11

**Authors:** Tobias Engeroff, Florian Delp, Daniel Niederer, David Groneberg

**Affiliations:** 1 Institute of Occupational, Social and Environmental Medicine, Goethe University Frankfurt am Main, Theodor-Stern-Kai 7, Frankfurt am Main, 60590 Germany; 2 Department for Preventive and Nutritional Medicine in Sports, Institute of Sports Sciences, Goethe University Frankfurt am Main, Ginnheimer Landstraße 39, Frankfurt am Main, 60487 Germany

**Keywords:** Ambient Particulate Matter Concentrations, Urban Running, Air Pollution, Respiratory Health, Mobile Exposure Monitoring

## Abstract

**Background:**

In urban environments, physical activity-induced health effects may be diminished by exposure to air pollution. Typical avoidance strategies, such as running through green spaces or avoiding major roads and rush hours, have not yet been adequately validated. This study quantified particulate matter exposure during running across three adjacent urban microenvironments: a major roadside, a residential area, and an urban park, accounting for diurnal variation.

**Methods:**

Mobile measurements of particulate matter with aerodynamic diameters ≤ 10 μm (PM_10_), ≤ 2.5 μm (PM_2.5_), and ≤ 1 μm (PM_1_) as well as local urban background concentrations of PM_2.5_ and PM_10_ were collected during 2 km runs (*n* = 60, pace 8:02–4:31 min/km). Runs on major roads (*n* = 20), residential streets (*n* = 20), or in an urban park (*n* = 20), as well as runs in the morning (*n* = 30) and in the early afternoon (*n* = 30), were equally distributed. Differences between routes, incorporating the effects of daytime and meteorological factors, on particulate matter concentrations were analysed using linear mixed-effects models.

**Results:**

The lowest concentrations across PM fractions occurred during early-afternoon runs in the park, whereas the highest exposure to PM_10_ across all environments was reached during morning runs on the same route. In line with the urban background levels, exposure across all routes was lower during early-afternoon than during morning runs. Humidity, air pressure, wind speed, and temperature were associated with particulate matter exposure during running. A sensitivity analysis using run-level means (*n* = 60) did not confirm the route or route×daytime effects as statistically significant, indicating that these patterns require confirmation in larger samples.

**Conclusion:**

Running in parks in the early afternoon and avoiding busy morning hours may help reduce fine and submicron particle exposure, particularly during calm, humid, and high-pressure conditions when pollutant dispersion is limited. ​However, park routes did not reduce, and at times increased, coarse-particle (PM_10_) exposure, especially in the morning. These route- and daytime-related differences were not confirmed in a run-level sensitivity analysis and should therefore be regarded as preliminary.

**Supplementary Information:**

The online version contains supplementary material available at https://doi.org/10.1186/s13690-026-02137-1.

**Table:** 

Text box 1. Contributions to the literature
• This study expands population health literature by providing direct, breathing-height mobile measurements of multiple particulate matter size fractions during aerobic exercise in distinct urban microenvironments.
• It suggests that time of day modifies spatial exposure risks, revealing that assumed “healthy” spaces like urban parks can yield higher coarse-particle exposure during busy morning hours.
• By analysing variation in PM₁ mass concentrations separately from PM₂.₅ and PM₁₀, the findings emphasize the necessity of analyzing submicron (PM_1_) fractions, in addition to broader background data, to more fully evaluate route-specific respiratory health risks for active urban populations.

## Background

Outdoor running is one of the most popular and most accessible health-enhancing physical activities [1]. Its beneficial effects on physical fitness and overall health are well documented [2]. However, performing outdoor activities in urban environments, particularly in areas and at times characterized by heavy traffic, may also entail health risks. Short-term exposure to traffic-related air pollution has been associated with airway and systemic inflammatory responses in healthy adults, and exercising in polluted air has been linked to adverse pulmonary and cardiovascular effects [3, 4]. For particulate matter with aerodynamic diameters ≤2.5µm (PM₂.₅), the overall evidence is considered consistent with a causal relationship with cardiovascular morbidity and mortality [5].

During aerobic exercise, ventilation rate increases substantially, leading to a higher inhaled dose of air and, consequently, also higher inhaled doses of airborne pollutants compared with resting conditions [6, 7]. In urban environments, particulate matter concentration and particle-size composition vary across locations and times of day. As motorized road traffic is a major local source through exhaust emissions, abrasion particles, and the resuspension of road dust, the highest concentrations particularly occur near traffic and during peak commuting periods [8].

Traffic-related particulate matter is a heterogeneous mixture of different constituents. Exhaust emissions mainly consist of combustion-derived particles such as black carbon and organic compounds, which are predominantly found in the submicron size range [9]. From a public health perspective, combustion-related constituents are of particular concern. Per unit mass, black carbon has shown larger associations with adverse health outcomes than PM₁₀ or PM₂.₅, although the associations were similar when expressed per interquartile range increase [9]. Long-term exposure to traffic-related air pollution has been associated with all-cause, circulatory and ischaemic heart disease mortality, with lung cancer mortality and with the onset of asthma in children and adults [10]. Because these constituents are concentrated in different size ranges, PM₁₀, PM₂.₅ and PM₁ mass concentrations measured near traffic partly reflect different sources.

Consequently, running near highly trafficked roads may increase the likelihood of exposure to high particulate matter concentrations. In response, runners habitually select routes perceived as healthier, most commonly green spaces and streets away from heavy traffic [11]. Although these settings seem to be healthier running environments, it remains unclear whether the particulate matter exposure in green spaces and streets away from heavy traffic is indeed lower when compared with urban roads, particularly when located within dense urban settings near high-traffic corridors. This assumption is further supported by a growing body of cycling research showing lower pollutant exposure on cycle paths and low-traffic routes compared with major roads [12–14]. Using the same mobile-monitoring approach applied here, we have previously shown that cyclists in Frankfurt am Main experience significantly lower PM exposure on major roads after road closure to motorized traffic [15]. Additionally, unpaved or gravel-covered paths, which some parks feature, have been linked to elevated coarse-particle concentrations through resuspension [16, 17], although this depends strongly on local surface type and pedestrian or cyclist activity rather than being an inherent property of green spaces. Residential streets with lower traffic intensity might therefore represent a further alternative, potentially combining reduced traffic emissions with more homogeneous paved surfaces.

A valid assessment of running-related particulate matter exposure requires measurements performed directly along running routes and ideally at breathing height, under realistic running speeds, and over exercise-relevant exposure durations. Urban background monitoring stations are feasible to assess temporal variations, but lack the option to evaluate spatial variations in particulate matter concentrations, especially for routes in proximity. Furthermore, runners may be exposed not only to different concentrations but also to different particle-size compositions. Local monitoring networks often do not assess the submicron particle fraction (PM₁) at all.

The three mass fractions differ in their public health relevance. PM₁₀ approximates the thoracic fraction that can pass the larynx, whereas smaller particles increasingly reach the unciliated airways and the alveolar region [18]. This distinction is relevant for runners, because the shift from nasal to oronasal breathing at higher ventilation rates bypasses part of the nasal filtration and increases the share of particles reaching the lower airways [3, 18]. Associations with mortality and cardiovascular morbidity are most consistently documented for PM₂.₅ [5], the evidence for the coarse fraction is less extensive and less consistent [19], and a growing number of studies links short-term PM₁ exposure to cardiorespiratory diseases [20]. Analysing PM₁₀, PM₂.₅ and PM₁ separately can therefore indicate whether route-related differences are driven by coarse particles from local mechanical sources or by finer, predominantly combustion-related particles of greater health concern.

Previous research on traffic-related exposure during active travel has mainly compared travel modes or contrasted on-road measurements with fixed-site data. Reviews of transport microenvironments report that concentrations close to traffic tend to be higher than in other everyday settings and are not adequately represented by fixed-site monitors, but most underlying studies examined commuting by car, bus, bicycle or on foot rather than recreational exercise [21, 22]. Cycling studies comparing route types found lower exposure on separated cycle paths and low-traffic streets than on major roads and identified traffic volume, traffic composition and road geometry as determinants of exposure [12, 23, 24], and a multi-microenvironment campaign in London reported a gradient from traffic intersections to parks [25]. Running-specific evidence remains scarce. Available studies either focused on PM₂.₅ in and around a single urban park [7] or on mass-participation events such as marathons [26], and the time of day has rarely been examined as a modifier of route-related differences. It therefore remains unclear whether the everyday choice between a park, a residential area and a major road within the same district translates into meaningful differences across particle-size fractions, and whether such differences persist across the day.

To address these gaps, the present study quantified potential differences in particulate matter exposure during running across three adjacent urban microenvironments: a major roadside, a residential area, and an urban park, accounting for diurnal variations and potential confounders. We hypothesized that the particulate matter concentration is the highest during morning roadside running and the lowest in an urban park during early-afternoon runs.

## Methods

### Trial design

This is a randomized crossover field trial with repeated measures. Measurements were performed on 10 separate days (weekdays only, to capture traffic- and commuting-related exposure patterns and to keep background activity levels comparable across measurement days) during November and December 2025. On each day, six runs were conducted during two time-blocks: three runs in the morning (9:00–12:00, covering the later part of the morning commuting period into the pre-noon hours) and three runs in the early afternoon (12:00–15:00). During each time-block, all three running routes were completed once, sequentially, not simultaneously, in randomized order (TE using randomizer.org for block randomization). Consequently, each route was performed equally often across all measurement days (allocation ratio 1:1:1 across environments and balanced across time points), resulting in a total of 60 runs. Running speed was standardized within a block of three runs and chosen to span an ecologically relevant range for amateur runners (8–4 min·km⁻¹). The speed assigned to each block was determined in a randomized order.

### Running routes

**Fig. 1:**
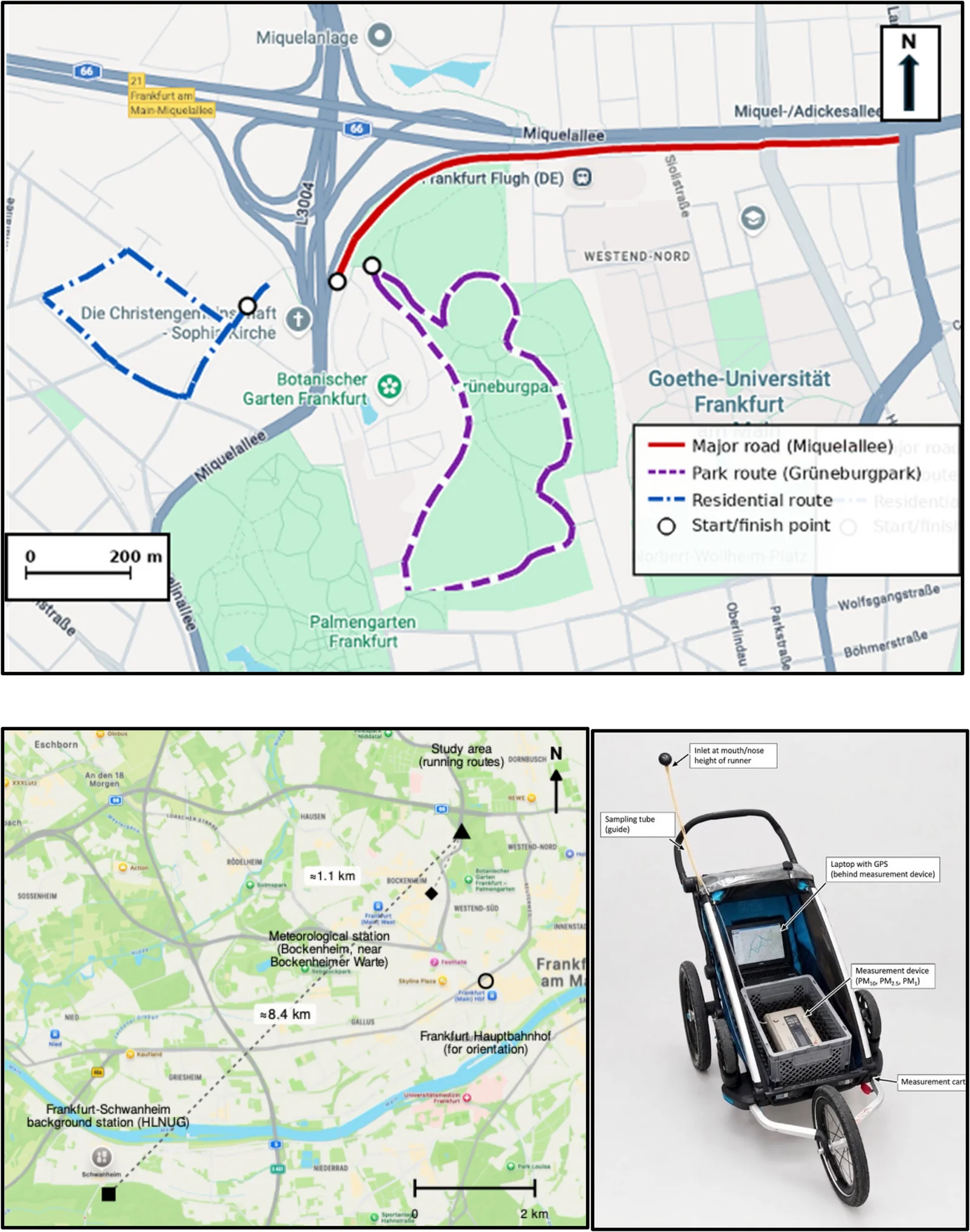
Overview of the three running routes, the running routes in relation to the air quality monitoring site (background station) an​d the meteorological station​, and the equipment (Mobile air pollution measurement system used during urban running)

The measurements were performed in Frankfurt am Main, Germany, a city with about 770,000 inhabitants and a population density of about 3,113 persons per km² that has enforced a low-emission zone covering nearly the entire urban area since 2008. Frankfurt Airport is located about 12 km southwest of the running routes. Aircraft engines are a major source of ultrafine particles, and measurements by the regional environmental agency around Frankfurt Airport showed markedly increased particle number concentrations downwind of the airfield and along the approach corridors, with the prevailing south-westerly winds carrying these particles towards the urban area [27]. Ultrafine particles, however, contribute very little to particle mass [27]. A substantial airport contribution to the PM₁₀, PM₂.₅ and PM₁ mass concentrations analysed here is therefore considered unlikely, whereas an influence on particle number concentrations, which were not analysed, cannot be excluded. Three outdoor running routes with comparable total distance (2 km) were selected: (1) Urban park/Green space with no motorized traffic; (2) Residential neighborhood with low traffic and moderate building density; (3) Major traffic road with multiple lanes and a wide shared pedestrian-cycle path. All routes were in the same city district. An overview of the routes is provided in Fig. 1.

The route along the major road is characterized by high motorized traffic density and represents an important urban transport corridor. The running course followed the pedestrian path directly adjacent to flowing traffic. Due to the constant vehicle flow, this route represents a typical high-emission urban environment with elevated traffic-related air pollution exposure.

The urban park route was located within a large inner-city green area adjacent to the urban transport corridor. The runs were performed on wide shared-use pedestrian and bicycle gravel paths separated from motorized traffic. The route passed through continuous green space with dense vegetation and tree coverage. Consequently, this route represents an urban environment with comparatively low particulate matter exposure from directly adjacent motorized traffic.

The residential street route was situated within a residential district with a comparable distance to the urban transport corridor. The course mainly followed side streets that were classified a priori as low-traffic based on street type (residential access roads rather than through-roads) and posted speed limits, rather than on quantitative traffic counts, with limited motorized vehicle flow. The surrounding environment consisted primarily of housing areas (Two to three-story buildings) with only occasional local traffic. This route represents air pollution exposure in a typical urban residential setting.

### Measurement equipment and outcomes

Exposure to airborne particulate matter during running was assessed using a portable Mini Laser Aerosol Spectrometer (Model 11-R, GRIMM Aerosol Technik GmbH, Germany). The device allows continuous real-time monitoring of aerosol concentrations. The instrument measures size-resolved particle concentrations in ambient air using a light-scattering measurement chamber and converts scattering signals into electrical output signals.

Particles in the size range of 0.25–32 μm were detected and classified into 31 size channels; in addition to the mass concentrations analyzed here, the instrument concurrently records particle number concentrations per channel, which were not used for the present analysis. Concentrations of particulate matter with aerodynamic diameters ≤ 10 μm (PM_10_), ≤ 2.5 μm (PM_2.5_), and ≤ 1 μm (PM_1_) were recorded simultaneously at 6-s intervals.

Before each measurement day, the device was calibrated according to the manufacturer’s specifications and connected to a laptop for data acquisition. To ensure temporal accuracy, the internal clocks of the aerosol spectrometer and the recording computer were synchronized prior to each measurement session.

During running, the instrument was transported in a running-compatible babyjogger/stroller. The inlet was positioned at approximately mouth height of the runner, allowing a realistic approximation of inhaled air exposure during exercise. To prevent vibration-related measurement artefacts, the device was placed in a foam enclosure and additionally secured with straps. A sampling tube was guided through an opening of the otherwise closed cabin of the stroller and mounted on a fixed rod attached to the frame. Figure 1 includes a picture of the setup. Throughout this manuscript, the term exposure refers to these ambient concentrations at breathing height along the routes and not to personal inhaled dose.

Mobile particulate matter measurements were interpreted in relation to urban background concentrations obtained from a stationary air quality monitoring station operated by the Hessian Agency for Nature Conservation, Environment and Geology (HLNUG) in Frankfurt-Schwanheim. The monitoring station is located approximately 8.5 km from the running routes. This station provides continuous measurements of PM₁₀ and PM₂.₅ within the Frankfurt metropolitan area. The air quality monitoring stations data provided half-hour average concentrations. No background monitoring data were available for PM₁. Therefore, PM₁ values were analysed as absolute concentrations only. The Frankfurt-Schwanheim station is classified as an urban background station [28] and is located at the urban fringe in an area used for recreation, about 110 m from the nearest road, and is therefore less affected by local traffic and industrial emissions compared to other available sites. Its data were used as a temporal rather than a spatial reference, that is, the half-hourly values entered the models to account for day-to-day and diurnal variation in background concentrations. They were not assumed to represent the absolute concentrations along the running routes. Particulate matter mass concentrations at urban background sites in the Rhine-Main area are of a similar magnitude [29]. Because this station is located closer to the airport than the running routes, airport-related emissions may influence its data [27, 29].

Meteorological information, including temperature [°C], relative humidity [%], wind speed [m/s], and atmospheric pressure [mmHg], were obtained from a single measuring station in the city district the running trials took place.

### Statistical analysis

For the statistical analyses, data from the mobile aerosol spectrometer were combined with particulate matter and meteorological data from the local monitoring stations and transferred into a single file. For consistency with the model-based estimates reported alongside them, descriptive statistics are reported as means and standard deviations (SD).

Linear mixed-effects models were fitted to account for the hierarchical structure of the data and the non-independence of observations. Route type (major road, residential street, park route) and time of day (morning vs. early afternoon) were included as fixed effects together with their interaction term. Measurement day was included as a random intercept to account for day-to-day variability and repeated measurement runs within each day. Meteorological variables (temperature, relative humidity, wind speed, and atmospheric pressure) were included as covariates to control for environmental influences on particulate matter concentrations. Urban background particulate matter concentrations were included as an additional predictor in the PM_2.5_ and PM_10_ models, including an interaction with daytime to account for temporal variation in background pollution levels. Background PM_1_ concentrations were not available and were therefore not included in the PM_1_ model.

Separate linear mixed-effects models were fitted for PM_1_, PM_2.5_, and PM_10_ concentrations. Model assumptions were evaluated by visual inspection of residual plots and assessment of the normal distribution of the residuals.

Post hoc, estimated marginal means were calculated to describe differences in particulate matter concentrations across route types and daytime. Model fit was evaluated using marginal and conditional pseudo-R² values. Because a large gap between marginal and conditional pseudo-R² can reflect a substantial contribution of the random-effects structure, we additionally report adjusted and conditional intraclass correlation coefficients (ICCs) for each model, following the variance-partitioning approach described in [30, 31]. As a further sensitivity analysis, we re-fitted route × daytime models on run-level means (one value per run, *n* = 60, using the same random-intercept-for-day structure) to assess whether conclusions were sensitive to the very large number of highly autocorrelated within-run observations (6-second sampling over ~ 13-minute runs) used in the primary models. We additionally examined running pace as a covariate at the run level and computed non-overlapping (PM_1–2.5_ and PM_2.5–10_) mass fractions directly from paired run-level readings. All statistical analyses were performed using IBM SPSS Statistics (Version 31). Statistical significance was defined as *p* < .05 for all tests.

## Results

A total distance of 131.2 km was covered at an average pace of 5:56 min/km (range 8:02–4:31 min/km) during 60 measurement runs. The average running time per run was 12:58 min, resulting in an overall recording time of more than 8 h and a final dataset comprising 6,881 observations for each particulate matter size fraction.

**Fig. 2:**
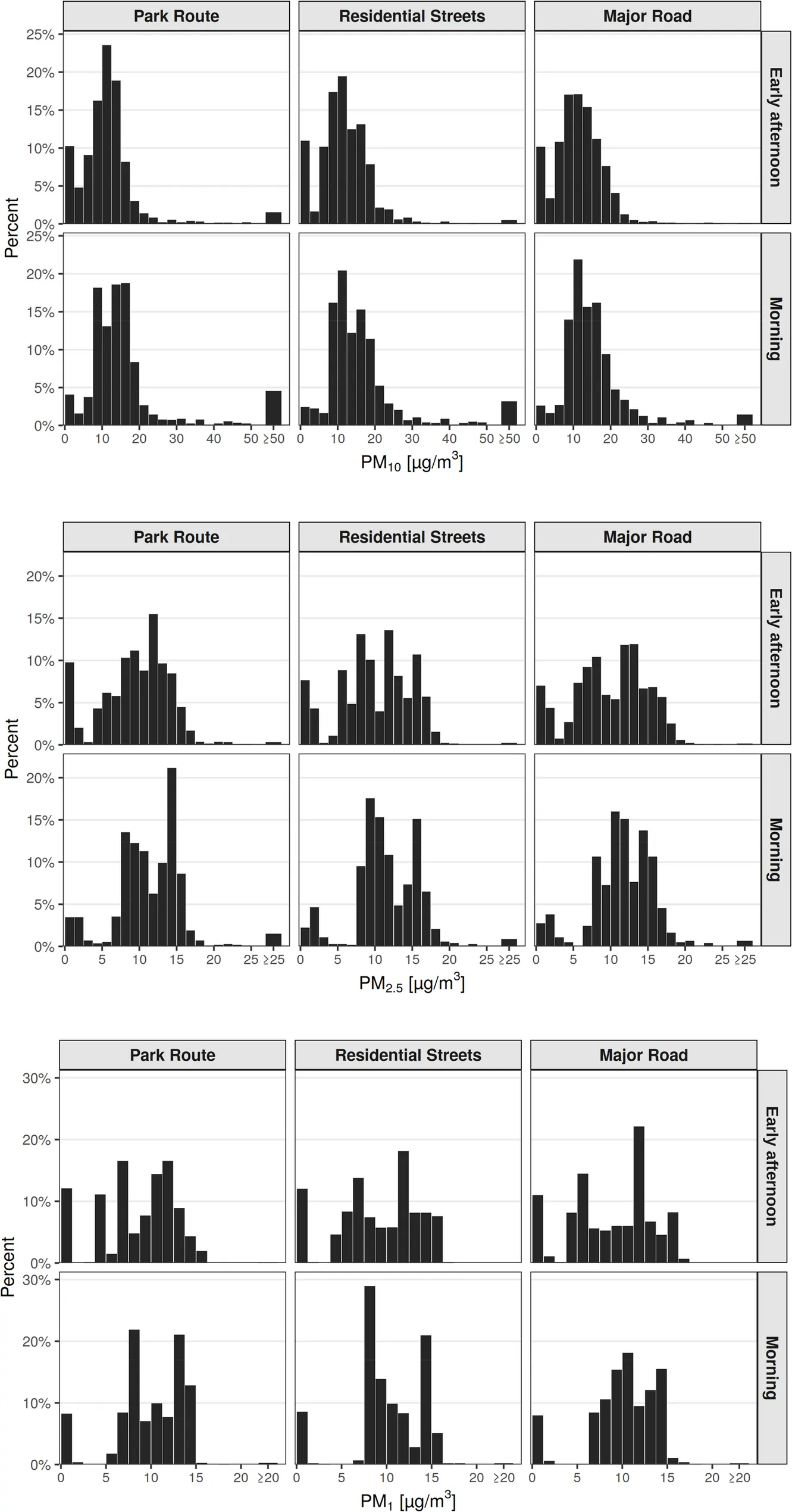
Percentage of all 6-second-interval readings within each route×daytime panel that fall into a given concentration bin (x-axis, µg/m³)

Descriptive statistics of urban background particulate matter concentrations, meteorological variables, and particulate matter exposure across running routes are summarized in Table 1. Figure 2 illustrates the percentage distribution of absolute personal exposure to airborne particulate matter during running across route types and daytime.

**Table 1:** Mean values and standard deviation for meteorological parameters, local urban background concentration and route specific exposure to PM_10_ and PM_2.5_ and PM_1_ separated for morning and early-afternoon time-blocks

Outcome	Route	Morning	Early afternoon
Urban Background PM_10_ [µg/m³]	Overall	14.0 ± 5.1	11.8 ± 5.8
Route Specific Exposure PM_10_ [µg/m³]	Park	19.2 ± 34.1	11.8 ± 10.1
	Residential streets	16.9 ± 15.6	11.8 ± 7.4
	Major road	15.4 ± 11.1	11.6 ± 6.8
Urban Background PM_2.5_ [µg/m³]	Overall	11.2 ± 4.8	9.6 ± 5.2
Route Specific Exposure PM_2.5_ [µg/m³]	Park route	11.7 ± 8.6	9.6 ± 4.9
	Residential streets	11.7 ± 6.1	10.0 ± 4.9
	Major road	11.8 ± 6.8	10.0 ± 4.9
Route Specific Exposure PM_1_ [µg/m³]	Park route	9.9 ± 3.8	8.5 ± 4.3
	Residential streets	10.1 ± 3.9	9.0 ± 4.4
	Major road	10.1 ± 3.7	8.9 ± 4.4
Temperature [°C]	Overall	4.4 ± 3.7	7.3 ± 4.1
Relative humidity [%]	Overall	83.7 ± 7.2	80.9 ± 6.7
Wind speed [m/s]	Overall	2.0 ± 0.8	1.9 ± 0.8
Air pressure [hPa]	Overall	1003.3 ± 6.6	1002.8 ± 6.5

The results of the models to examine the influence of route type and time of day on particulate matter exposure and estimated marginal means are including full variance components together with adjusted and conditional ICCs, are reported for each model in Table 2. Adjusted ICCs were 0.357 (PM_10_), 0.773 (PM_2.5_) and 0.994 (PM_1_); conditional ICCs were 0.270, 0.611 and 0.934, respectively. The particularly high values for PM_1_ indicate that, once the fixed effects are accounted for, observations sharing the same measurement day remain highly correlated – consistent with the high temporal resolution of the mobile measurements (6-second intervals over runs lasting approximately 13 min), which yields many non-independent readings per run rather than 60 statistically independent observations. Route and daytime effects and their standard errors, which are the primary quantities of interest, remain valid under this random-effects specification irrespective of how the variance is partitioned.

**Table 2:** Model-based estimates with standard errors and confidence intervals as well as statistical values for personal exposure to airborne particulate matter during running (μg/m^3^), PM_2.5_ (μg/m^3^) and PM_10_ (μg/m^3^). Estimated marginal means of personal exposure to particulate matter during running by route type and daytime

	Estimate	Standard error		t-value	*p*-value			95% CI Lower Level	95% CI Upper Level	
PM_10_ [μg/m³]	Pseudo R² (marginal): 0.245; Pseudo R² (conditional): 0.515									
Intercept	-1019.697	326.112		-3.127	0.003			-1672.079	-367.314	
Park route (ref: Major road)	4.632	0.697		6.643	<0.001			3.265	5.999	
Residential streets (ref: Major road)	0.918	0.706		1.300	0.194			-0.466	2.301	
Early afternoon (ref: Morning)	0.093	1.615		0.058	0.954			-3.075	3.262	
Park route × Early afternoon	-4.365	0.932		-4.685	<0.001			-6.192	-2.539	
Residential streets × Early afternoon	-0.558	0.950		-0.587	0.557			-2.421	1.305	
Temperature [°C]	0.510	0.353		1.444	0.150			-0.185	1.206	
Relative humidity [%]	1.832	0.133		13.749	<0.001			1.570	2.095	
Wind speed [m/s]	2.285	3.694		0.619	0.540			-5.227	9.797	
Air pressure [hPa]	0.886	0.335		2.649	0.010			0.216	1.556	
Early afternoon × background PM_10_	-0.572	0.119		-4.787	<0.001			-0.806	-0.337	
Morning × background PM_10_	-0.655	0.126		-5.198	<0.001			-0.902	-0.407	
PM_2.5_ [μg/m³]	Pseudo R² (marginal): 0.210; Pseudo R² (conditional): 0.821									
Intercept	-543.626	116.169		-4.680	<.001			-771.927	-315.324	
Park route (ref: Major road)	0.264	0.209		1.262	0.207			-0.146	0.675	
Residential streets (ref: Major road)	-0.041	0.206		-0.197	0.844			-0.444	0.363	
Early afternoon (ref: Morning)	-0.299	0.458		-0.654	0.513			-1.196	0.598	
Park route × Early afternoon	-0.910	0.270		-3.365	<.001			-1.440	-0.380	
Residential streets × Early afternoon	0.490	0.275		1.779	0.075			-0.050	1.029	
Temperature [°C]	1.008	0.124		8.141	<.001			0.764	1.252	
Relative humidity [%]	0.714	0.045		15.822	<.001			0.625	0.803	
Wind speed [m/s]	-0.036	1.473		-0.025	0.980			-2.951	2.878	
Air pressure [hPa]	0.487	0.120		4.062	<.001			0.251	0.723	
Early afternoon × background PM_2.5_	0.572	0.054		10.609	<.001			0.466	0.678	
Morning × background PM_2.5_	0.554	0.053		10.409	<.001			0.449	0.658	
PM_1_ [μg/m³]	Pseudo R² (marginal): 0.060; Pseudo R² (conditional): 0.994									
Intercept	-761.598	36.877		-20.653	<0.001			-833.888	-689.308	
Park route	-0.567	0.063		-9.006	<0.001			-0.690	-0.443	
Residential streets	0.143	0.062		2.299	0.022			0.021	0.265	
Early afternoon	0.202	0.092		2.209	0.027			0.023	0.382	
Park route × Early afternoon	-0.338	0.081		-4.190	<0.001			-0.496	-0.180	
Residential streets × Early afternoon	0.310	0.082		3.758	<0.001			0.148	0.471	
Temperature [°C]	-0.112	0.033		-3.359	<0.001			-0.178	-0.047	
Relative humidity [%]	0.384	0.014		26.849	<0.001			0.356	0.412	
Wind speed [m/s]	-7.054	0.486		-14.526	<0.001			-8.006	-6.102	
Air pressure [hPa]	0.761	0.038		19.999	<0.001			0.686	0.835	

Estimated marginal means of personal exposure to particulate matter during running by route type and daytime										
Route	Daytime	PM_1_			PM_2.5_			PM_10_		
		Mean	SE	95% CI	Mean	SE	95% CI	Mean	SE	95% CI
Park route	Early afternoon	11.16	0.64	9.91–12.41	14.46	0.77	12.91–16.02	13.03	1.21	10.64–15.41
Park route	Morning	12.56	0.73	11.13–13.99	15.48	0.89	13.70–17.27	17.40	1.32	14.81–19.98
Residential streets	Early afternoon	11.89	0.65	10.61–13.16	15.56	0.83	13.90–17.22	15.00	1.27	12.51–17.49
Residential streets	Morning	12.60	0.75	11.12–14.07	15.18	0.97	13.23–17.13	15.80	1.39	13.07–18.53
Major road	Early afternoon	12.29	0.68	10.95–13.63	15.11	0.81	13.49–16.73	17.04	1.24	14.61–19.47
Major road	Morning	12.56	0.79	11.01–14.11	15.22	0.93	13.35–17.10	17.04	1.37	14.35–19.73

*Abbreviations*: *PM* particulate matter, *SE* standard error, *CI* confidence interval

For PM_10_, the routes differed (F(2, 2653.476) = 13.633, *p* < .001), whereas the main effect of daytime was not significant (F(1, 1389.611) = 1.004, *p* = .317). A route × daytime interaction was observed (F(2, 6849.201) = 13.149, *p* < .001), indicating that route-related differences varied depending on the time of day. Compared with the reference category (major road), park runs were associated with higher PM_10_ concentrations (β = 4.632, *p* < .001), whereas no difference between residential street and major road routes was found (β = 0.918, *p* = .194). The significant interaction term showed that PM_10_ concentrations on park routes were lower in the early afternoon (β = −4.365, *p* < .001), while no daytime effect was observed for residential street routes. Estimated marginal means showed variation across route–daytime combinations (Table 2). Lower PM_10_ exposure levels were observed for early-afternoon runs on park routes, whereas morning runs on major road and park routes tended to be associated with higher concentrations.

For PM_2.5_, the mixed-effects model showed an effect of route type (F(2, 875) ≈ 3.28, *p* = .038), whereas the main effect of daytime was not significant (F(1, 5068) ≈ 1.03, *p* = .310). A route × daytime interaction was observed (F(2, 6346) ≈ 13.56, *p* < .001). Model coefficients indicated lower PM_2.5_ concentrations on park routes in the early afternoon compared with the reference condition (β = −0.910, *p* < .001), while residential street routes showed a tendency toward higher concentrations in the early afternoon (β = 0.490, *p* = .075). Estimated marginal means ranged from approximately 14.46 to 15.56 µg/m³ across route–daytime combinations (Table 2). The lowest estimated exposure levels were observed on park routes in the early afternoon, whereas higher concentrations were observed on residential street routes in the early afternoon and major road routes during morning runs.

For PM_1_, the mixed-effects model revealed effects of route type (F(2, 6869) ≈ 40.7, *p* < .001), daytime (F(1, 6866) ≈ 4.9, *p* = .027), and their interaction (F(2, 6869) ≈ 17.6, *p* < .001). Compared with the reference category (major road route), running on park routes was associated with lower PM_1_ concentrations (β = −0.567, *p* < .001), whereas residential street routes showed slightly higher concentrations (β = 0.143, *p* = .022). The interaction term indicated that PM_1_ concentrations differed between routes depending on the time of day, with lower concentrations on park routes during early-afternoon runs (β = −0.338, *p* < .001) and higher concentrations on residential street routes in the early afternoon (β = 0.310, *p* < .001). Estimated marginal means further supported these patterns (Table 2), indicating generally lower PM_1_ exposure on park routes, particularly during early-afternoon runs, whereas higher concentrations were observed on major road routes, especially during morning runs.

Meteorological variables significantly influenced particulate matter concentrations. Relative humidity and air pressure showed positive associations across all particulate matter categories. Higher temperature was associated with higher PM_2.5_ but lower PM_1_ concentrations. Furthermore, PM_1_ was lower during runs with higher wind speeds.

As a sensitivity analysis, we additionally re-analyzed the data at the level of run means (*n* = 60, one value per run, using the same random-intercept-for-day structure as the primary models). In this analysis, neither the route main effect (joint Wald tests: PM_10_ *p*=.923, PM_2.5_ *p*=.989, PM_1_ *p*=.958) nor the route×daytime interaction (likelihood-ratio tests: PM_10_ *p*=.806, PM_2.5_ *p*=.838, PM_1_ *p*=.912) reached statistical significance for any particulate matter fraction. The daytime effect for PM_10_ was statistically significant when day was modelled as a fixed blocking factor (*p*=.004) but not when modelled as a random intercept (*p*=.196), as in the primary analysis. Daytime effects for PM_2.5_ and PM_1_ were not significant under either specification. This indicates that the route and route×daytime effects reported above for the primary (high-frequency) models are not confirmed when the unit of replication is restricted to the 60 independent runs, and that their statistical significance in the primary analysis is attributable in part to the very large number of non-independent within-run observations. Although running speed was randomized, pace differed between morning and early-afternoon blocks (5.5 vs. 6.4 min·km⁻¹, t = − 2.69, *p*=.009). It was independently associated with run-level PM_10_ (*p*=.009) after adjustment for route and daytime, an association not evaluated in the primary models. Non-overlapping PM_2.5–10_ and PM_1–2.5_ mass fractions, computed directly from paired run-level readings, showed a similar morning-versus-early-afternoon coarse-particle pattern to that seen in the nested-fraction results above (Supplementary Table S1).

## Discussion

### Principal results and hypotheses verification

The specific running route and the time of day were both associated with particulate matter exposure during running. Because route, surface material, vegetation, traffic, and pedestrian activity co-varied by design, “route” in this study is best understood as a composite of these unmeasured, co-located factors rather than evidence for any single causal mechanism. We note that the main effect of daytime was observed for PM_1_ whereas the daytime main effect was not significant on its own for PM_10_ and PM_2.5_. Daytime influence on these two fractions is captured only through its significant interaction with route (i.e. daytime mattered conditionally on route, not uniformly across routes). Interactions of route and daytime indicate that route-related benefits are not consistent throughout the day. The park route showed reduced overall particulate matter exposure only in the early afternoon and consistently lower concentrations of the submicron particles PM_1_ across the day. However, PM_10_ exposure reached the highest level across all route–daytime combinations during morning runs in the park. For most particle-size fractions, we did not observe consistently lower mass concentrations on residential streets compared with those on major road routes. However, this does not establish equivalent traffic-pollution exposure overall, since pollutants we did not measure (e.g. elemental carbon or ultrafine particle number) could still differ meaningfully between routes even where PM mass concentrations are similar. Meteorological factors influenced exposure. Stable high-pressure conditions were associated with increased overall particulate matter concentrations. Higher temperatures were linked to higher PM_2.5_ but lower PM_1_ concentrations. Furthermore, higher wind speeds led to lower PM_1_ exposure. Our hypothesis – that particulate matter concentration would be highest during morning roadside running and lowest in the park in the early afternoon – was therefore only partly confirmed since the lowest exposure across all three particle-size fractions consistently occurred during early-afternoon park runs, but the highest exposure was not consistently observed for morning major-road running. For PM_10_ specifically, the highest concentration occurred on the park route in the morning, opposite to our hypothesis. For PM_1_ and PM_2.5_, morning concentrations on residential streets were marginally higher than on the major road. We interpret this as partial, fraction-specific support for our hypothesis rather than full confirmation. Moreover, a run-level sensitivity analysis did not confirm the route main effect or the route×daytime interaction as statistically significant for any fraction, indicating that the route- and interaction-related findings reported above should be regarded as preliminary and motivating further study with a larger number of independent runs, rather than as firmly established effects.

### Implications for route choice

Contrary to the assumption that parks provide lower particulate matter exposure [11], the highest PM₁₀ concentrations were observed on the park route during morning runs. Our findings on PM₁₀ concentrations can likely be explained by the local generation of coarse particles, which exhibit greater spatial variability due to faster sedimentation [32–34]. One plausible, though not directly tested, explanation is higher pedestrian and cyclist activity combined with the gravel surface of the park route, which could resuspend coarse dust particles and contribute to short-term PM₁₀ peaks [16, 17]. The observed increases in PM₁₀ might be primarily driven by coarse particles, which were not accompanied by increases in smaller alveolar-deposited fractions. We did not directly measure particle morphology, mineral composition, or resuspension flux, so this source attribution remains an inference rather than a demonstrated mechanism. As larger particles predominantly deposit in the upper respiratory tract rather than penetrating deeply into the lungs, the health impact and relevance of elevated PM₁₀ exposure during morning park runs may be lower than those associated with finer particles.

The assumption that finer particles in the PM_2.5_ range are less dominated by very local sources and more strongly influenced by broader urban background sources, such as emission processes, is consistent with our observation that the variation in PM_2.5_ was considerably smaller across the investigated routes. Fine particulate matter is largely generated by traffic-related combustion (~ 26%), followed by secondary sulfate (~ 23%) and nitrate (~ 16%), which are formed in the atmosphere through the oxidation of SO₂ and NOₓ and their subsequent conversion, with additional contributions from refuse incineration (~ 15%) and soil dust (~ 13%) [17]. In contrast to larger particles, PM₂.₅ penetrates the lower respiratory tract and deposits in the alveolar region. Consequently, PM₂.₅ has been associated with a range of adverse cardiopulmonary health outcomes and increased mortality, although these health impacts depend strongly on its chemical composition and sources [35]. Given the health implications, even the modest route- and daytime-related differences observed in our results may have implications for primary prevention. Despite the small variability across routes, the park route resulted in lower PM₂.₅ exposure during the early afternoon, when the area was presumably less intensively used by active commuters and background concentrations were lower. Under these conditions, the mitigating effects of vegetation and the greater distance from traffic emissions [33] may become more apparent, whereas the impact of local emission sources may become pronounced. A comparable multi-microenvironment mobile campaign in London reported a gradient from traffic-intersection over street-canyon to park for all PM fractions [25], pointing in the same direction as our park-route findings for PM_1_ and early-afternoon PM_2.5_. Contrastingly, our PM_10_ results diverge from this pattern during morning hours, which we attribute to the park route’s unpaved surface and pedestrian or cyclist activity rather than to a general property of green space. This interpretation is supported by earlier studies indicating significantly lower PM exposure when major roads are closed to motorized traffic [15] and our observation that residential street routes were associated with higher PM₂.₅ exposure during the early afternoon, which may be influenced by localized sources such as residential traffic or delivery vehicles. Overall, the data align with cycling research showing that traffic volume, composition, and road geometry independently shape personal air pollution exposure [23], that pollution and noise exposure can vary substantially even within similarly classified urban roads [24], while cycle paths reduce exposure relative to major roads [12, 13].

In line with earlier studies reporting comparatively limited variation in ultrafine particle number concentrations between traffic conditions during cycling [36], we similarly found low variance in our mass-based PM₁ concentration between morning and early-afternoon runs on major roads. In contrast to PM₂.₅, route choice appeared to play a more pronounced role for PM₁, as higher concentrations observed during major road runs likely reflect the strong contribution of traffic-related combustion emissions, which are a major and predominantly local source of submicron particles [37]. In addition, nucleation and condensation processes in traffic plumes can promote the formation of very small particles near busy roads, whereas such processes are less pronounced in park environments due to lower precursor concentrations and reduced direct traffic influence. The bigger route-related differences observed for PM₁ compared with PM₂.₅ underline the importance of analyzing submicron particles when evaluating the impact of route choice during health-enhancing physical activities such as running. From a prevention perspective, the distinction is particularly relevant, as smaller particles can penetrate deeply into the respiratory system and may differ in their health impacts compared with PM₂.₅, with several studies suggesting that ultrafine particles, a distinct, number-based size class below approximately 0.1 μm that our optical instrument could not resolve, could be particularly harmful [16, 20]. Although PM_1_ mass concentration is at best a partial and imperfect proxy for ultrafine particle number, and the two should not be equated, the smaller particles are of particular interest because they can penetrate further into the bronchioles and alveolar region, where prior work links deep lung deposition to systemic inflammatory and cardiovascular responses [16, 20]. This is why we analyzed PM_1_ as a separate fraction rather than relying on PM_10_ or PM_2.5_ alone, even though, as noted above, it cannot be equated with ultrafine particle number.

### Impact of meteorological conditions

Meteorological conditions significantly influenced particulate matter concentrations in our measurements. Relative humidity and air pressure showed positive associations across all particulate matter fractions. Alongside genuine atmospheric behavior, hygroscopic particle growth is a plausible contributor to the strong association with humidity. Measurements were conducted during the transition from autumn to winter in Germany, and our data indicate stable atmospheric conditions, which are known to favor pollutant accumulation near the surface by suppressing vertical mixing [38, 39]. Our results indicate that these meteorological factors might have a greater impact on local concentrations of coarse particles than on finer particle fractions, particularly when inversion layers limit pollutant dispersion [40].

In line with earlier studies [23, 41], our data indicate that fine particle (PM_2.5_) concentrations were positively associated with higher temperatures, whereas PM₁ concentrations decreased with increasing temperature. The opposing effects may reflect temperature-dependent changes in particle formation and size distribution. Higher temperatures can enhance secondary aerosol formation and promote particle growth through condensation and coagulation processes, shifting particle mass from submicron particles toward larger fine particles. Lastly, higher wind speeds were associated with lower submicron (PM_1_) concentrations in our data, consistent with enhanced dispersion and dilution of fine and ultrafine particles, as also observed in previous mobile monitoring studies [42, 43].

### Limitations

A key strength of this study is the rigorous repeated-measures design under real-world conditions, allowing a detailed representation of the spatial variability [12] in exposure experienced during running across different urban route types.

Although simultaneous multi-fraction mobile monitoring has previously been performed in other cities [25], this study is among the first studies to report PM₁₀, PM₂.₅, and PM₁ exposure experienced during running across different urban route types. Furthermore, the simultaneous measurement of multiple particle size fractions (PM₁₀, PM₂.₅, and PM₁) enabled a more differentiated analysis of exposure patterns, particularly revealing greater route-related differences in the submicron particle fraction. Measurements were conducted during the transition from autumn to winter, a period often characterized by stable atmospheric conditions, reduced dispersion, and increased emissions from traffic and residential heating, allowing the investigation of exposure under comparatively high urban particulate matter levels. However, this seasonal focus may limit the generalizability of the results to other seasons. This study compared two distinct time periods, capturing temporal variations in both traffic-related PM exposure and the impact of green-space use by pedestrians and cyclists. Future studies should extend this approach to additional time periods (e.g., early morning, evening, and nighttime) to further assess the influence of exercise timing on PM exposure. The following limitations should be acknowledged and addressed in future research. First, the urban background station used for comparison was located approximately 8.5 km from the running routes, providing 30-minute averages which were not independently validated. Consequently, future studies should include assessments for minute-scale background variability at the study location and co-located background references. Second, although the morning block was intended to capture commuting-related traffic, it extended to noon, and we did not directly measure traffic volume, so morning concentrations cannot be assumed to reflect peak rush-hour traffic specifically. Third, running pace was standardized within each three-run block and randomized across blocks but varied considerably between morning and early-afternoon runs due to the small number of assessment days. A post-hoc run-level check indicated that pace differed between morning and early-afternoon blocks and was independently associated with PM_10_, although route and daytime effects remained non-significant with or without adjustment for pace. With 60 runs across 10 days, the design offers limited statistical power to detect smaller route- or daytime-related differences. Future studies should adopt a design with a considerably large range of running paces (8:02–4:31 min·km⁻¹) and include more assessment days to enable sufficient analysis of the effect of running pace. Furthermore, the inclusion of participants would lead to the option to assess ventilation, heart rate, and other physiological outcomes with the aim of comparing the impact of intensity-related changes in ventilation and exposure duration. Finally, although this study reports, to our knowledge, among the first mobile running-based measurements of PM_1_, PM_2.5_ and PM_10_ conducted in Frankfurt am Main, comparable simultaneous multi-fraction mobile monitoring has previously been performed in other cities [25], and the value of our data lies primarily in a running-specific comparison rather than in the multi-fraction approach itself. We also note that the plausibility of pedestrian- or cyclist-driven gravel resuspension as a source of the elevated morning PM_10_ on the park route is not self-evident given the modest number of runners and other path users typically present during ordinary recreational use (in contrast to, for example, a mass-participation event such as a marathon); ambient wind-driven resuspension and disturbance by other park users (e.g. dog walkers, cyclists) not captured in our protocol may also have contributed, and we did not measure park usage levels directly, so this explanation remains speculative. We also did not record wind direction, which the meteorological station did not provide. Given that the major-road corridor lies mostly to the north of the park and residential routes (Fig. 1), wind direction could plausibly influence route-specific concentrations, and we consider the missing wind direction a clear limitation of this study. Regional wind direction from the meteorological station at Frankfurt Airport could serve only as an indicative proxy, as its distance of about 12 km and its open airfield setting would not reflect the local flow conditions within the built-up study district. We therefore strongly recommend that future studies record wind direction locally along each route. In addition, only particle mass concentrations were assessed, while the local differences in chemical composition of particulate matter need to be further analyzed [44, 45].

## Conclusions

Our study suggests that route choice and time of day are associated with particulate matter exposure during urban running, although a run-level sensitivity analysis did not confirm the route and route×daytime effects as statistically significant, so these associations should be interpreted as preliminary. The magnitude of these effects varies across particle size fractions. Running in parks and avoiding busy morning hours on weekdays may help reduce exposure to submicron and, to a lesser extent, fine particles, particularly under meteorological conditions that limit pollutant dispersion. In contrast, residential street routes did not consistently provide lower exposure levels across the investigated particulate matter fractions. While PM₂.₅ showed a less pronounced spatial pattern, submicron particles (PM₁) exhibited clearer route-related differences. When interpreting potential health implications, it should be considered that the observed concentrations on urban roads and streets remained within current air quality guideline ranges. Thus, the health benefits of regular physical activity are likely to outweigh the potential risks from particulate matter exposure, particularly where access to green running routes is limited.

## Supplementary Information

Supplementary Material 1.

## Data Availability

Data and materials are available upon reasonable request. The datasets generated and analyzed during the current study are available from the corresponding author.

## Abbreviations

CI: Confidence interval
HLNUG: Hessian Agency for Nature Conservation, Environment and Geology
ICC: Intraclass correlation coefficient
PM: Particulate matter
SD: Standard deviation
SE: Standard error
SPSS: Statistical Package for the Social Sciences
