# Supplementary material for: Air pollution exposure during urban running in Frankfurt, Germany: a randomized crossover comparison of particulate matter levels on park, roadside, and residential routes across morning and early afternoon hours
Source: Arch Public Health. 2026 Nov 11;84:244. doi: 10.1186/s13690-026-02137-1 (PMC13753208; doi:10.1186/s13690-026-02137-1)
Supplement: Supplementary file 1 [file 13690_2026_2137_MOESM1_ESM.docx]

# **Supplementary Material**

*Manuscript: “Air Pollution Exposure During Urban Running in Frankfurt, Germany: A Randomized Crossover Comparison of Particulate Matter Levels on Park, Roadside, and Residential Routes Across Morning and Midday Hours”*

**Table S1. Non-overlapping particulate matter mass fractions by route and daytime, computed directly from paired run-level readings.**

| **Route** | **Daytime** | **n** | **PM2.5–10 (µg/m³) Mean ± SD** | **PM1–2.5 (µg/m³) Mean ± SD** |
| --- | --- | --- | --- | --- |
| Major road (Miquelallee) | Morning | 10 | 2.91 ± 2.03 | 1.42 ± 0.42 |
| Major road (Miquelallee) | Midday | 10 | 1.38 ± 1.11 | 1.11 ± 0.59 |
| Park route (Grüneburgpark) | Morning | 10 | 3.54 ± 5.36 | 1.57 ± 1.11 |
| Park route (Grüneburgpark) | Midday | 10 | 1.09 ± 1.03 | 0.96 ± 0.50 |
| Residential route | Morning | 10 | 3.69 ± 4.68 | 1.37 ± 0.63 |
| Residential route | Midday | 10 | 1.27 ± 1.30 | 0.98 ± 0.53 |

*PM2.5–10 = PM10 − PM2.5; PM1–2.5 = PM2.5 − PM1, each computed per run from paired route-specific (personal) exposure means before averaging across runs within each route×daytime cell (n = 10 runs per cell; 60 runs in total). Unlike the nested (cumulative) PM10, PM2.5 and PM1 fractions analysed in the main mixed-effects models, these coarse (PM2.5–10) and intermediate (PM1–2.5) fractions are non-overlapping by construction and were computed from raw run-level means rather than from model-adjusted estimates, since the primary models include fraction-specific covariates and are therefore not directly comparable across fractions (see main text). One of 60 runs showed a small negative PM2.5–10 value (− 0.1 µg/m³), consistent with measurement noise rather than a violation of the nested mass relationship.*

*Referenced in the Results (sensitivity analysis) and in the response to Reviewer 1, Major Comment 5.*
